# Prediction of gas velocity in two-phase flow using developed fuzzy logic system with differential evolution algorithm

**DOI:** 10.1038/s41598-021-81957-3

**Published:** 2021-01-27

**Authors:** Meisam Babanezhad, Samyar Zabihi, Iman Behroyan, Ali Taghvaie Nakhjiri, Azam Marjani, Saeed Shirazian

**Affiliations:** 1 Institute of Research and Development, Duy Tan University, Da Nang, 550000 Vietnam; 2 Faculty of Electrical-Electronic Engineering, Duy Tan University, Da Nang, 550000 Vietnam; 3 Department of Artificial Intelligence, Shunderman Industrial Strategy Co., Tehran, Iran; 4 Department of Process Engineering, Research and Development Department, Shazand-Arak Oil Refinery Company, Arak, Iran; 5 Faculty of Mechanical and Energy Engineering, Shahid Beheshti University, Tehran, Iran; 6 Department of Computational Fluid Dynamics, Shunderman Industrial Strategy Co., Tehran, Iran; 7 Department of Petroleum and Chemical Engineering, Science and Research Branch, Islamic Azad University, Tehran, Iran; 8 Department for Management of Science and Technology Development, Ton Duc Thang University, Ho Chi Minh City, Viet Nam; 9 Faculty of Applied Sciences, Ton Duc Thang University, Ho Chi Minh City, Viet Nam; 10 Laboratory of Computational Modeling of Drugs, South Ural State University, 76 Lenin Prospekt, Chelyabinsk, 454080 Russia

**Keywords:** Mathematics and computing, Computational science, Computer science, Process chemistry, Theoretical chemistry

## Abstract

In this investigation, differential evolution (DE) algorithm with the fuzzy inference system (FIS) are combined and the DE algorithm is employed in FIS training process. Considered data in this study were extracted from simulation of a 2D two-phase reactor in which gas was sparged from bottom of reactor, and the injected gas velocities were between 0.05 to 0.11 m/s. After doing a couple of training by making some changes in DE parameters and FIS parameters, the greatest percentage of FIS capacity was achieved. By applying the optimized model, the gas phase velocity in x direction inside the reactor was predicted when the injected gas velocity was 0.08 m/s.

## Introduction

A major role is played by liquid/gas systems in a variety of chemical engineering sectors. One of the most renowned technical equipment is bubble columns reactors which are usually used for contacting between two gas and liquid phases. Bubble columns are normally applied for slow kinetic reactions like oxidation, alkylation, hydrogenation, hydroformylation, decarbonization, Fischer–Tropsh synthesis, desulfurization, and fermentation^1,2^.

Wastewater treatment sites, producers of organic acids, yeasts, and cell cultures utilize these reactors in their processing sites^3^. The form and shape of these columns are simple, with no moving element. In addition, these reactors are featured with economical operation costs, easy maintenance, and desirable mass/heat transfer flux. As the disadvantages, complicated hydrodynamics deeply depend on the geometry and flow velocities. In addition, local/global parameters (flow pattern, phase velocities, gas phase hold-up, turbulence, and bubble size) have a direct and complicated effect on the design variables.

Bubble column (BC) reactors are usually utilized in industrial work like heterogeneous churn turbulent flow pattern^4^. Thereby, it is imperative to examine such columns under such operational circumstances for better performance and optimization. There have been many empirical correlations developed to design such systems. Such correlations normally possess a limited validity domain as to operating conditions, geometries, or physical properties. There is a need to develop new computation models to simulate bubble column reactors with a wider validity range compatible with both homogeneous/heterogeneous flow patterns.

For years, the only way of scaling up bubble columns was through macroscopic equations to elaborate on hydrodynamics of system^5,6^. Currently, however, computational fluid dynamics (CFD) technique is employed as a reliable procedure to find local/global properties through bubbly flows^7^ and eliminate the limits of the conventional scale-up method through costly experimental measurements.

To function effectively, bubble column reactors need efficacious liquid—gas phases mass, momentum, and energy transfers. While bubble column reactors have simple design, the modelling is not easy. Operational and design parameters of the column, which are related to each other in a complicated manner, dictate phase velocity, gas volume fractions, bubble size, flow regime, and turbulence. There are unmet needs with models to simulate bubble column reactors for homogeneous/heterogeneous flow patterns. The CFD simulation might meet the requirements for studying large bubble columns in an extensive range of operating circumstances.

It is possible to divide multiple flows into dispersed and separate flows and BCs function in a dispersed regime. Dispersed flow is featured with bubbles of gas and the continuous liquid flow. Finding good results using high volume fraction in the dispersed phase is not easy. To explain the way such multiphase system works, different methods can be followed and among them, Euler–Lagrange and Euler–Euler (E–E) methods are the most commonly applied ones.

Many researchers have used Reynolds-averaged Navier–Stokes (RANS) calculation for Navier–Stokes equations to model these systems by E–E approach that describes the two phases as inter-penetrating continua^8,9^. There is a need to accurately model the interactions between the continuous and disperse phases^7^. Such interactions are controlled by a variety of interfacial forces^10,11^, and among them drag force is the most important. Additionally, we need a great volume of computation power to handle the complicated multiphase simulations using the CFD. Thus, while the novel experimental techniques and CFD tool measure fluid velocity with high accuracy, given the limitations of these methods like high cost, complicated engineering problems, difficult implementation, lengthy process, and the like, using the intelligent method can be an answer to the complicated problems like predicting the bubbly flows in reactors^12–17^. There are several AI techniques proposed by studies like fuzzy logic^18^, neural networks, neuro-fuzzy, or artificial bee colony (ABC) algorithm and differential evolution (DE) algorithm^19–22^. One of the notable artificial intelligence methods is the fuzzy logic introduced by Zadeh^23^. In contrary to the standard set theories, the model assumes that expression of membership is not limited to 0 or 1. As stated by the fuzzy logic, a member is able to be a part of a cluster in disparate membership degress. Uncertainty appears in many life events. The fuzzy inference system enables us to interpret the unspecific circumstances as rules using the decision-making mechanism. Thus, it can be utilized to solve different physical and chemical problems^24–29^.

The numerical method of solving for the fluid motion in the reactor could take much time, and in the processes that optimization is needed, this time extends. Also, when the fluid flow in a reactor or a domain has two or several phases or the flow is heterogeneous or turbulent, it could bring lots of complexities with itself. This means that the numerical method for solving becomes very difficult because of choosing the boundary conditions, the convergence area, and the limitations of the numerical method. So, the solving needs computer resources with high ability, meaning that we need high-performance computers and clusters to have parallel runs. Due to all the difficulties, AI algorithms could carry out a part of the responsibility to complete the numerical method for solving. Moreover, in most cases, the numerical method for solving is repetitive due to the high ability and speed of algorithms. The algorithms can numerically complete the solving from the CFD data, and they could speed up the optimization process as well as the whole numerical method for solving the process. On the other hand, among AI algorithms, the DE algorithm has not frequency used in predicting flow pattern with CFD datasets. There is this possibility to test and use this algorithm more in this field; therefore, we can check its potentiality in training and prediction when it is used in the fuzzy system. Apart from using the DE method in predicting flow distribution and optimization of physical processes, there is still a lack of information about tuning this model for the best selection of datasets and model parameters. There are still many questions and barriers with regards to the best way of training continuous datasets (such as results of numerical calculation of partial or ordinary differential equations), and then predict them, or optimize the processes. The behavior of this AI method can be completely different when it faces with non-discrete datasets. In this regard, this model needs to be fully modified with regards to model parameters, selection of datasets, and the number of training datasets for a higher level of prediction accuracy and capability. Due to connectivity between input and output dataset and meaningful relation between input/output data patterns, they can be evaluated by local pattern recognition. In this case, the pattern of AI dataset can be compared with a continuous dataset (such as CFD dataset), and we can examine the overall behavior of the model based on the local prediction dataset^30^. However, with the existing conventional statistical assessment, we can test the overall model accuracy and prediction capability. The proper selection of datasets and model parameters in a continuous framework can be a robust modeling way for other datasets that are calculated with numerical methods.

In previous studies, the DE method beside fuzzy system has been used widely as an AI algorithm or machine learning method. The method has a high potentiality in the prediction of physical problems^31–33^. Due to its high potentiality in prediction, the method has been used in various fields. So, in the current study, we use this method for learning of the CFD data, and after that, we complete the decision and prediction processes via the fuzzy inference system. We specifically used the DE algorithm for the training of the CFD data. Furthermore, after the training process, DE and fuzzy logic algorithms were used for the prediction process. It is worth mentioning that in this process, we used fuzzy logic for an exact prediction. Indeed, we used the data from the numerical method for solving in a reactor with 2 phases, i.e. gas and liquid, and our purpose is to create the training with the DE domain and examine the potentiality of the DE algorithm in training. Differential evolution algorithm (DE) is used as a trainer in FIS to predict data that was extracted from simulation of a 2D two-phases reactor, including gas phase and liquid phase. By considering some data as the inputs and output, learning processes were done. Moreover, we trained information, by data that were extracted from a situation in which the injected gas velocity from bottom of the reactor was 0.05 and 0.11 (m/s), then we predicted gas phase velocity in x direction when the injected gas velocity was 0.08 (m/s).

The DE model is used to predict the flow characteristics in the bubble column reactor and this model is also compared with other AI methods for further assessments, such as the ant colony optimization method and ANFIS method. In addition to this analysis, we specifically developed the DE model to predict continuous datasets with regards to tuning model parameters and data selection.

## Methodology

### CFD approach

Here, Euler–Euler (E–E) multiphase method is employed to evaluate the average mass, energy, and flow equations separately for each phase along with the volume fraction equation. Indeed, E–E procedure treats individual phase as interpenetrating continua, thereby volume fractions are taken as space and time function and their sum equals to 1. The equation of E–E is given as follows^34,35^:

Continuity equation^35^:

1 $$\frac{\partial }{{\partial {\text{t}}}}({\uprho }_{{\text{k}}} {\upvarepsilon }_{{\text{k}}} ) + \nabla ({\uprho }_{{\text{k}}} {\upvarepsilon }_{{\text{k}}} {\text{u}}_{{\text{k}}} ) = 0.$$

To compute the momentum equation, all interfacial forces (e.g. drag, turbulent dispersion, lift, vertical mass, and wall lubrications) are combined. Equation (2) describes the momentum transfer^35^:

2 $$\frac{\partial }{{\partial {\text{t}}}}({\uprho }_{{\text{k}}} {\upvarepsilon }_{{\text{k}}} {\text{u}}_{{\text{k}}} ) + \nabla .({\uprho }_{{\text{k}}} {\upvarepsilon }_{{\text{k}}} {\text{u}}_{{\text{k}}} {\text{u}}_{{\text{k}}} ) = { } - \nabla .({\upvarepsilon }_{{\text{k}}} {\uptau }_{{\text{k}}} ) - {\upvarepsilon }_{{\text{k}}} \nabla {\uprho } + {\upvarepsilon }_{{\text{k}}} {\uprho }_{{\text{k}}} {\text{g}} + {\text{M}}_{{{\text{I}},{\text{K}}}} .$$

The following equation interprets the stress term of bubbles and liquid phase as ^35^:

3 $${\uptau }_{{\text{k}}} = - {\upmu }_{{{\text{eff}},{\text{k}}}} (\nabla {\text{u}}_{{\text{k}}} ) + (\nabla {\text{u}}_{{\text{k}}} )^{{\text{T}}} - \frac{2}{3}{\text{I}}(\nabla. {\text{u}}_{{\text{k}}} ).$$

where ${\upmu }_{{{\text{eff}},{\text{k}}}} { }$ stands the effective viscosity. Detailed description of the models’ equations are reported elsewhere^8,16,36–39^.

### Takagi, Sugeno, and Kang, fuzzy inference system (FIS)

In prior works, the fuzzy controller and types of fuzzy inference system have been fully explained to optimize the processes in nature or presented as a problem solver. For instance, Takagi, Sugeno, and Kang have designed the structure of FIS, which translate the conceptual understanding of human in making decisions^40–42^. This type of structure can be coupled to other learning formats for better decision in the physical processes that human needs computational calculation for several problems. Different learning framework can learn the dataset with different algorithms and data randomization selection within the frame of fuzzy. These learning processes can also have a different level of model accuracy or training time. In the current study, we used different algorithms to investigate the ability of each of them separately. Also, the ability of training, as well as the ability of the decision in the fuzzy logic system were combined to prediction the model. We employed DE method to train the system, and after that, the data were used fuzzy logic for the prediction. For better comparing the accuracy of the model, we used ANFIS, or ACO to complete the training. After the methods were combined with the fuzzy logic system, we can have our model for the purpose of prediction. We completed the training from all of the obtained models in the form of iterative in AI, and the iterative part of the model is called an iteration. After solving for the iterative according to the convergence and error criteria, we stop the system for solving the iterative. The data in training and the intelligence in the training process were combined with the fuzzy logic system to provide the predictions.

The antecedents and membership functions are very identical with the Mamdani FIS structure, while the polynomial consequent can be used and replaced with fuzzy framework. In addition, a Mamdani FIS structure can be observed as a 0-th order TSK FIS. The TSK rule framework can be described, such as following^43^:

4 $${\text{R}}_{{\text{i}}} {\text{: IF}}\;{\text{ x}}_{{1}} \;{\text{is }}\;{\text{A}}_{{{\text{i1}}}} \;{\text{and}}\; \ldots \;{\text{and}}\;{\text{x}}_{{\text{n}}} \;{\text{is}}\;{\text{A}}_{{{\text{in}}}} \;{\text{THEN }}\;{\text{y}} = {\text{C}}_{{{\text{i1}}}} *{\text{x}}_{{1}} + \cdots {\text{ C}}_{{{\text{in}}}} *{\text{x}}_{{\text{n}}} + {\text{C}}_{{{\text{in}}}} + {1}$$

One of the main abilities over the Mamdani model is about small number of rules in the main structure of the model. On the other hand, we can distinguish between various FIS structures based on the weighted average of the rule output parameters rather than the max operator mechanism^44^. This model behavior and connection in the FIS structure can be observed in the framework of TSK. Additionally, the rule output can provide less computational cost and efforts due to defuzzification calculation in the model of AI. This model is also known as Sugeno model^43^.

One of the commonly used popular computing frameworks is the Takagi, Sugeno, and Kang (TSK) fuzzy inference system (FIS), which is based on theory of fuzzy set, fuzzy reasoning, and if–then rule. This framework has been effectively used in areas like data classification and expert systems. In terms of fuzzy reasoning, Takagi and Sugeno introduced if–then rules for construction of FIS architecture^45^. Here, x direction, y direction and injected gas velocity are assumed as FIS inputs to achieve gas phase velocity in x coordinate as FIS output.

For each input parameter fuzzy process generates the behavior of membership function as a function of input parameters that defines the connectivity between input parameters and the complexity of parameters within the domain of fuzzy rules. Input selection and associated dataset for each input during learning can be fully coupled with number of membership functions or types of function that describes the degree of membership functions in each input. w_i_ can also be calculated in the model and show the rule strength of the ith rule R_i_. w_i_ can be described for different input parameters, such as $X, \;Y$ and $V_{g}$ and written as^46^:

5 $${\text{w}}_{{\text{i}}} = {\upmu }_{{{\text{Ai}}}} ({\text{X}}){{ \upmu }}_{{{\text{Bi}}}} ({\text{Y}}){\upmu }_{{{\text{Ci}}}} ({\text{V}}_{{\text{g}}} )$$

where μ_Ai_, μ_Bi_ and μ_Ci_ explain signals from implemented membership functions (MFs) on inputs, x coordination (X), y coordination (Y) and injected gas velocity (V_g_). In Eq. (5), each input parameter, such as location of computing nodes and gas velocity, can be defined in the training mode.

The relative firing strength in each rule is achieved and the fuzzy-model output f_i_ is computed by a weighted mean(WM) defuzzification as follows^15^:

6 $$\overline{{{\text{w}}_{{\text{i}}} }} {\text{f}}_{{\text{i}}} = \overline{{{\text{w}}_{{\text{i}}} }} ({\text{p}}_{{\text{i}}} {\text{X}} + {\text{q}}_{{\text{i}}} {\text{Y}} + {\text{r}}_{{\text{i}}} {\text{V}}_{g} + {\text{s}}_{{\text{i}}} )$$

where p_i_, q_i_, r_i,_ and s_i_ are defined as the if–then rules' parameters known as consequent parameters. The signals are aggregated to yield the output of model, and represent the estimation result. With the aim of updating the parameters, a hybrid learning algorithm is employed where gradient descent technique updates the MFs parameters and Least Square Estimate (LSE) tecnique updates consequent factors.

### Differential evolution (DE) algorithm

Price and Storn^47^ developed differential evolution (DE) algorithm as a breakthrough algorithm. It is developed for global optimization problems of continuous domains with 3 control search parameters, i.e.:

1. F: mutation control parameter, which is for control of the extent to which the differential variation is amplified.
2. CR: the crossover control parameter as a constant parameters to determine what parameter associates with what trial vector parameter in the crossover operation.
3. NP: the size of population, which is the number of individuals in the population.

Several studies were conducted on DE and its applications^46–51^ and recommended value ranges for F, CR, and NP^50^. These factors indicate if the algorithm is able to find a near-optimum solution effectively or not. Adopting the right value using trial–error approach takes a large amount of time^52,53^. There are several studies on the effect of these parameters on the performance of DE^52^.

By adding tolerances as two novel parameters and taking the diversity of the population into account, we can adjust the amounts of the mutation control parameter and the crossover control parameters to achieve a higher algorithm efficiency and improve the quality of solution^49^. In^54^, a self-adaptive method was proposed to estimate DE parameters, the crossover parameters, and the mutation amplification. The way these 3 control parameters affect the DE performance was illustrated by^48^ by performing experiment on test functions. Consequently, new solutions to improve the effectiveness, robustness, and efficiency were found by adopting better approaches to set the DE’s search control parameter values.

### Geometrical structure

In this work, a cylindrical-based bubble column with height and diameter of 162 cm and 10 cm, respectively was simulated. The column also features two nozzles of 0.9 cm diameter and 5 cm higher than the bottom plate of the column. The liquid–gas dispersion was heated by an electrical heater and the superficial gas velocity was equal to 0.05 m/s.

### Boundary conditions and numerical methods

The simulations were done in ANSYS-Fluent on the basis of superficial gas velocity, the gas velocity from each sparger orifice was calculated. The bubble column outlet is featured with degassing boundary condition. Non-slip and free slip boundary conditions are implemented at the wall edge, on the liquid and gas bubbles, respectively. These conditions are offered by former studies^54–57^. In practice, the bubbles are under no fraction from the wall and it can travel along the boundary wall with no limitation. Therefore, it is assumed that there are almost not contact between the bubbles^58^.

Here, the control volume method was used to discretize the conservation equations. Generally, the flow field can be obtained using a variety of solution procedures like finite difference^59,60^, Lattice Boltzmann^60–64^, and finite volume method^38,64–67^. The most reliable and robust technique, which was used by CFX is finite volume discretization. This approach is capable of yielding the single/multiphase flow, and heat/mass transfer within an arbitrary geometry with or without structured grid^38,64–69^. The technique has been used by several studies to obtain the flow regime and gas dynamics through the reactor^38,39,65,66,70^. It is possible to solve the equation system using the SIMPLEC procedure. Because of the decrement in numerical diffusion and dispersion in the Eulerian framework, it is possible to employ the Total Variation Diminishing (TVD) in the numerical method^65,66,70–73^.

Bubbling flow computation is done for 1400 s, and all CFD studies used time step of 0.1, which was determined based on the reality that the maximum courant-Friedrichs-Levy (CFL) number should be smaller than 1. Some reports are existed that with CFL < 1, the numerical method gives accurate predictions of the multiphase features and better refinement of time step does not result in notable changes of the flow pattern result. In addition, CFL > 1 leads to inaccurate predictions^13,74–80^.

## Results and discussion

Here, DE algorithm is applied in the FIS training step in order to reach the best FIS Capacity. The number of extracted data is 2000 that was obtained when the injected gas velocity from bottom of the reactor was 0.05 and 0.11 (m/s). Each data consists of characteristics of fluids including x and y direction, injected gas velocity (FIS inputs), and gas phase velocity in x direction (FIS output). The amount of data applied in the training step is 65% and the maximum FIS iteration is 150 and also the type of clustering is subtractive clustering. Changes in some parameters like the number of inputs and cluster influence range (CIR), which is a parameter of FIS and the number of population, which is a parameter of DE algorithm were examined. To begin we did the learning processes with one input and CIR = 0.5, 0.4, 0.3 and 0.2 by considering number of population = 4, 8, 12, and 16 for each CIR, respectively. Results in Table S1, Appendix, show that when the number of input is 1 changes in amount of CIR and number of population had not any considerable changes in the amount of RMSE error for training and testing steps, and the lowest amount of RMSE for training is 0.00192 and for testing is 0.000194 in another word, percentage of FIS Capacity is about 29%.

To reach an enormous capacity of prediction, the second FIS input was considered. By repeating training as well as testing steps for a diversity of CIR and number of population, the amount of RMSE error declined to 0.000127 for training process and 0.000132 for testing process which indicates that we achieved 77% of intelligence. This significant positive change in FIS Capacity suggests that increasing the number of inputs has the maximum positive effect on FIS Capacity in comparison with other variable parameters (see Table S2, Appendix).

The best FIS Capacity was achieved that is 99.9% by adding injected gas velocity as third input and repetition of training and testing steps for a different range of CIR and the number of population. Table S3 (Appendix) indicates the amount of RMSE for training process declined to 5.2E−06 and for testing process declined to 7.2E−06. This amount was obtained when CIR = 2 and number of population was 16.

All initial and tuning parameters for DEFIS, ACOFIS, and ANFIS method are described in Table 1. This table shows that the number of input parameters for all methods is identical (number of inputs = 3), and the percentage of training data is 65% of whole datasets. In the FIS section, the clustering type and FIS type is Subtractive Clustering and Sugeno, respectively. In the Subtractive Clustering section, more effective tuning parameters can be selected. In this part, CIR, type of input membership function, type of output membership function, Squash factor, Accept and Reject ratio are 0.2, guassmf, linear, 1.25, 0.5, and 0.15, respectively. Tuning parameters that have a big impact on the model are selected in the next stage, which are specific model parameters for each prediction model. For the DEFIS model, the number of population, Crossover Probability, and the number of the rule are 16, 0.5, 64, respectively. However, in the ACOFIS method, different parameters are used in the model, such as the number of ants = 10, pheromone effect = 0.5, and the number of rules equal to 64. In the final stage in the ANFIS method, only a number of rules can be designed in the model, and it is 65. For the final stage of modeling parameters, all parameters are similar. In this regard, the maximum of training iteration, Error Goal, Initial Step Size, Step Size Decrease and Step Size Increase is 150, 0, 0.01, 0.9, and 1.1, respectively.

**Table 1:** DEFIS, ACOFIS, ANFIS set parameters for learning processes.

Method		DEFIS		ACOFIS		ANFIS	
FIS parameters	Number of inputs	3		3		3	
	Number of P (%)	65		65		65	
	Clustering Type	Subtractive clustering		Subtractive clustering		Subtractive clustering	
	FIS type	Sugeno		Sugeno		Sugeno	
Subtractive clustering parameters	Cluster influence range (CIR)	0.2		0.2		0.2	
	Type of input membership function	guassmf		guassmf		guassmf	
	Type of output membership function	Linear		Linear		Linear	
	Squash factor	1.25		1.25		1.25	
	Accept ratio	0.5		0.5		0.5	
	Reject ratio	0.15		0.15		0.15	
Algorithms important parameters		Number of population	16	Number of ants	10	–	–
		Crossover probability	0.5	Pheromone effect	0.5	–	–
		Number of rule	64	Number of rule	64	Number of rule	65
Training options	Maximun of training iteration	150		150		150	
	Error goal	0		0		0	
	Initial step size	0.01		0.01		0.01	
	Step size decrease	0.9		0.9		0.9	
	Step size increase	1.1		1.1		1.1	

**Figure 1:**
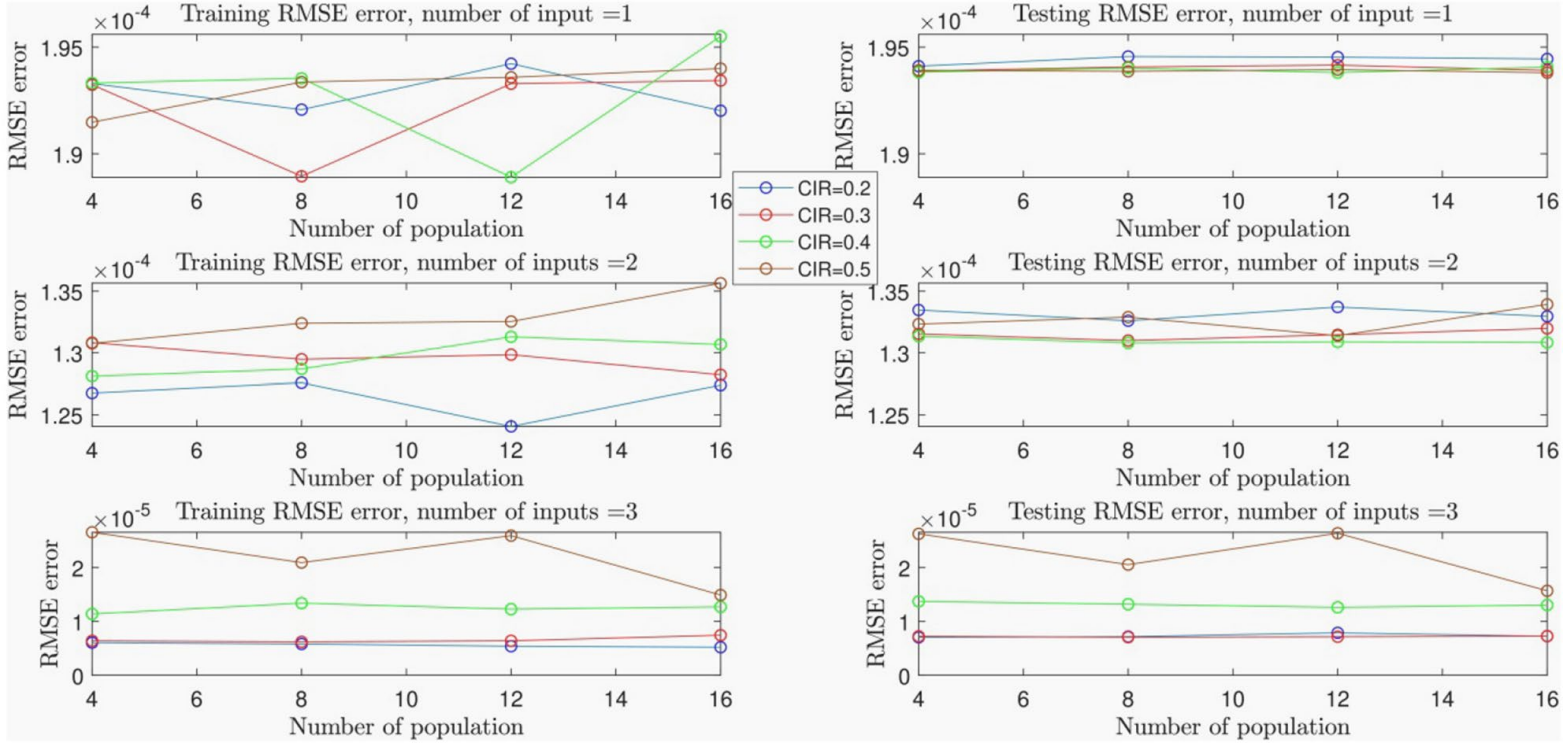
DEFIS learning processes with changes in the number of population as DE algorithm parameter and cluster influence range (CIR) as subtractive clustering parameter.

Figure 1 illustrates the effects of population, the number of CIR, and the number of input on the RMSE error of the system. We also consider the error in the testing/training steps. As shown, when the number of input is one, that is a very low number; a meaningful effect is not seen for the number of CIR and the number of population in both testing and training processes. Nevertheless, by increasing the number of inputs, the system reaches a significant intelligence, and the effect of the number of CIR becomes meaningful. Moreover, the effect of the number of population on RMSE error improves in both testing and training. For instance, Fig. 1 for number of inputs = 3 shows when the number of CIR is low, including 0.2, and 0.3, the error is in its lowest amount. This shows that the system has a very low amount of error, as well as a low CIR number. After studying the RMSE error, we fixed the system with the lowest RMSE error, which is the best system regarding the statistics and error. After that, we study the system in regression and pattern recognition domains to see how the system can predict our model.

**Figure 2:**
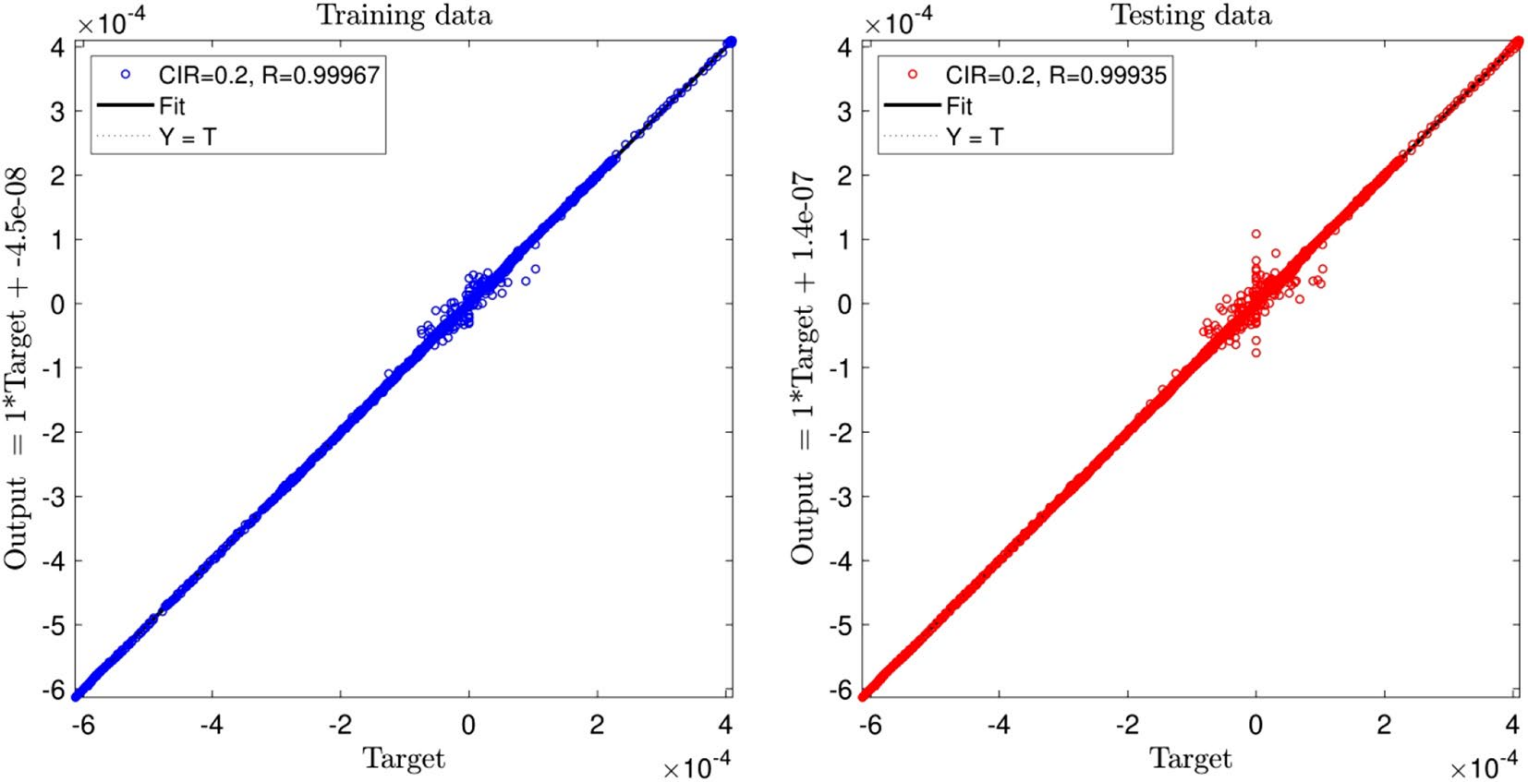
DE training and testing processes when CIR is 0.2, and number of input is 3.

**Figure 3:**
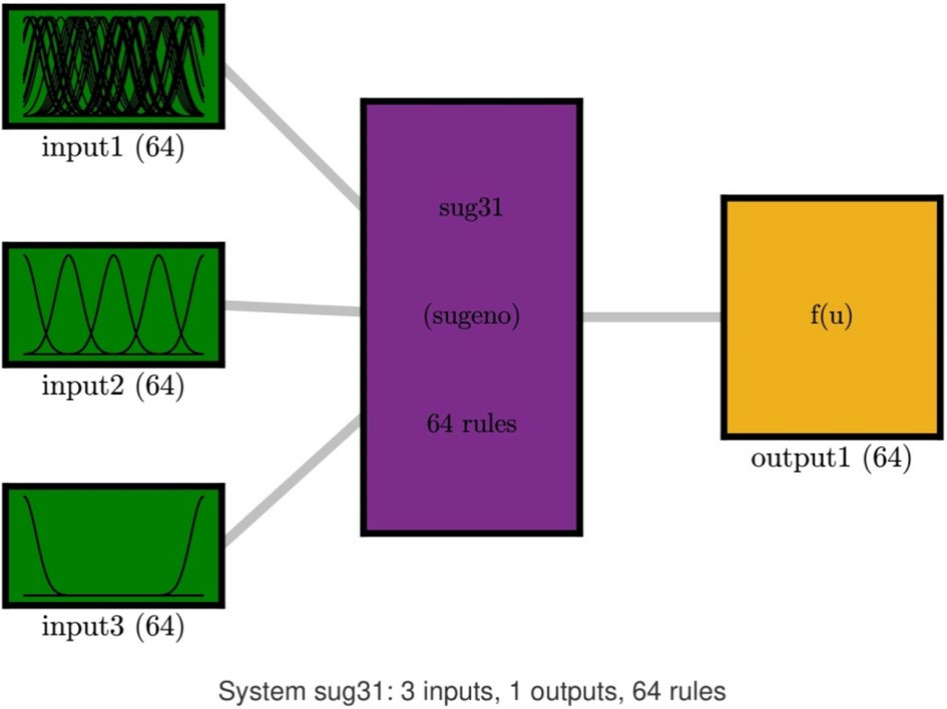
FIS system schematics when number of input is 3, and number of rule for each input membership function is 64.

Figure 2 shows that the value of regression (R^2^) for both training and testing processes is more 99.9%. According to the FIS structure number of rules is 64 for each input, hidden layer of FIS and output equally which is depicted in Fig. 3.

**Figure 4:**
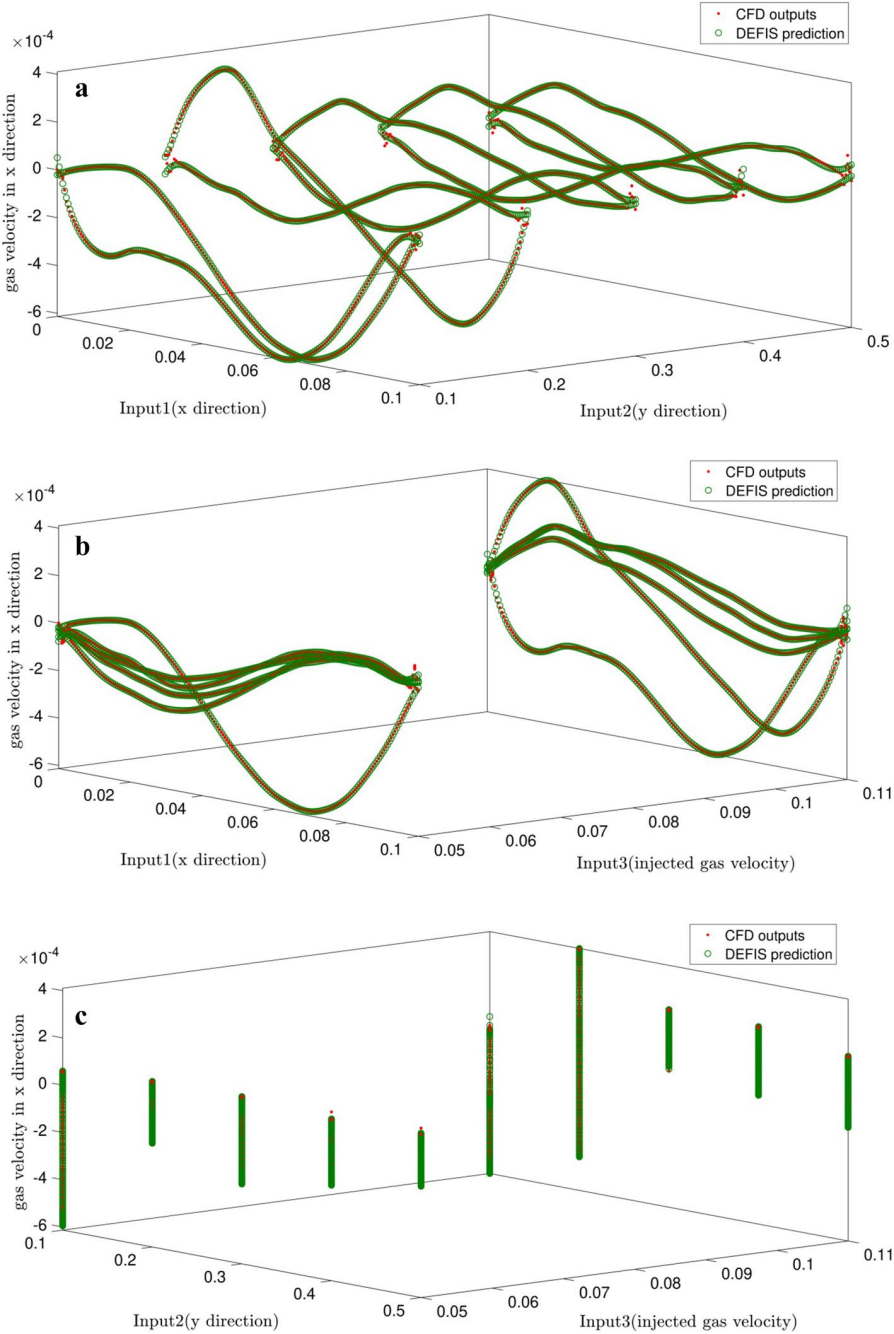
(**a**) Comparison of gas velocity between CFD output and DEFIS prediction with considering first and second inputs. (**b**) Comparison of gas velocity between CFD output and DEFIS prediction with considering first and third inputs. (**c**) Comparison of gas velocity between CFD output and DEFIS prediction with considering second and third inputs.

**Figure 5:**
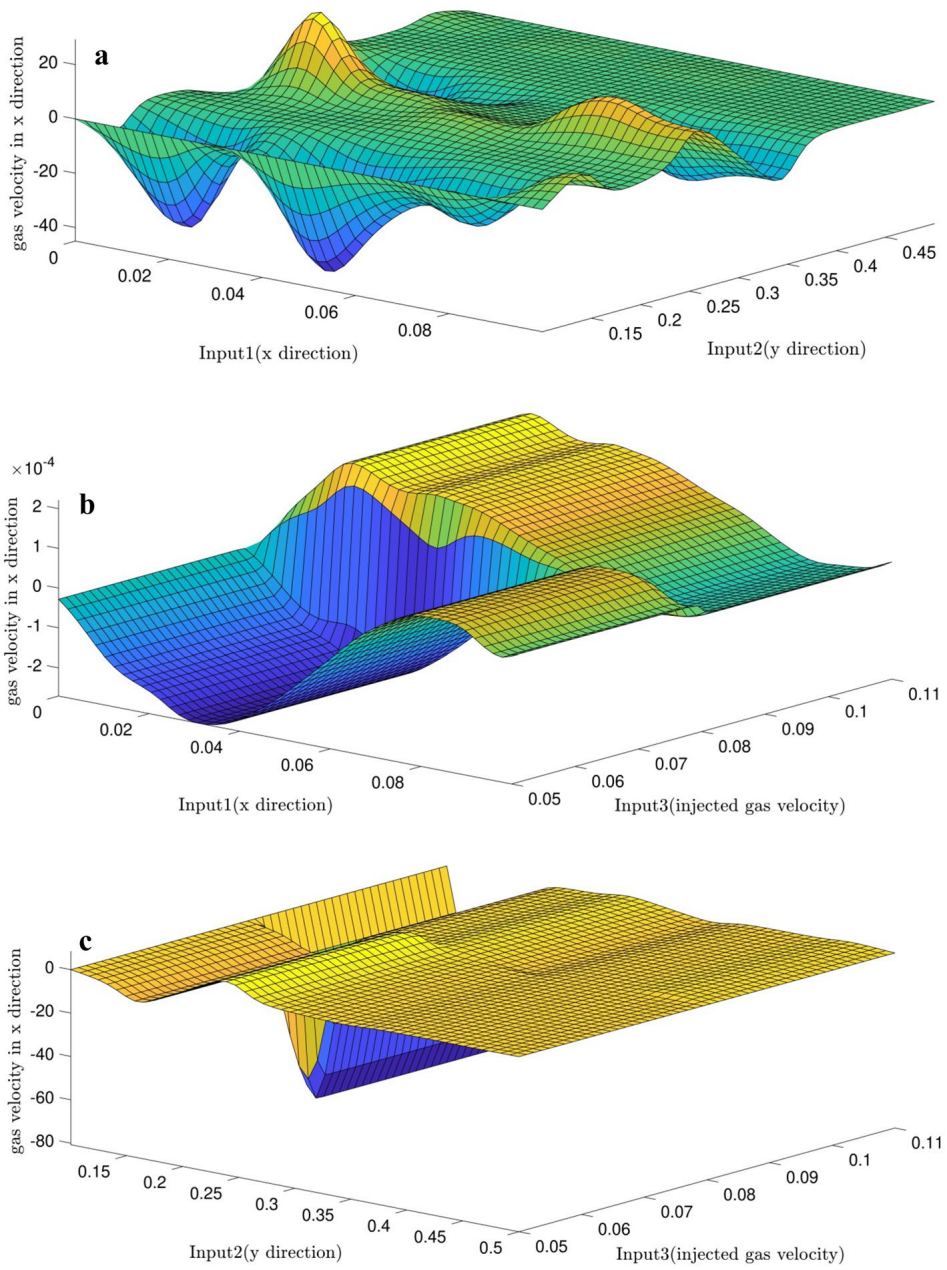
(**a**) Gas velocity predicted surface with considering first and second inputs. (**b**) Gas velocity predicted surface with considering first and third inputs. (**c**) Gas velocity predicted surface with considering second and third inputs.

For intelligence validation, data involved in the learning step are predicted and compared with the CFD results, there is a good adaptation between them and it is depicted in Fig. 4a–c. Moreover, by employing FIS structure, surfaces are predicted based on the inputs which are depicted in Fig. 5a–c. By implementing 2 of the inputs, gas phase velocity in x direction as the FIS output can be extracted via predicted surfaces obtained in Fig. 5.

**Figure 6:**
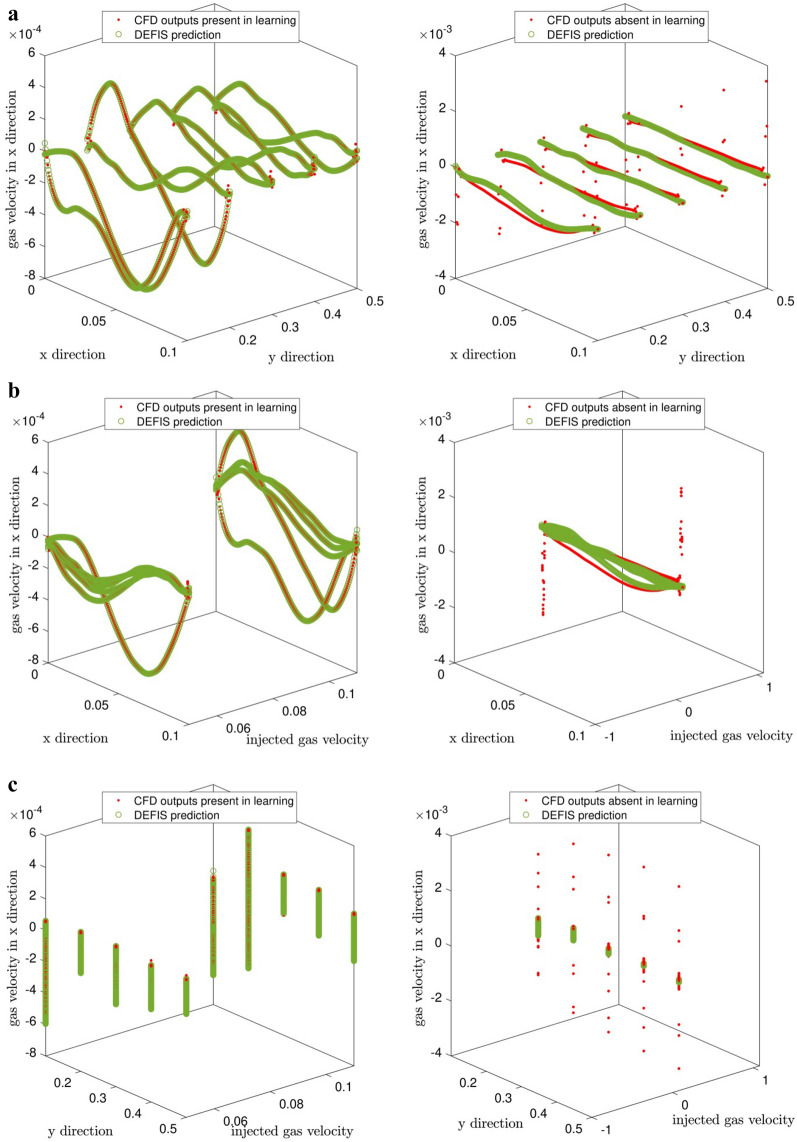
(**a**) Comparison of predicted gas velocity and CFD output (gas velocity) which is present in DEFIS learning (in left figure) and comparison of predicted gas velocity and CFD output (gas velocity) which is absent in DEFIS learning (the right figure) (with considering inputs 1 and 2). (**b**) Comparison of predicted gas velocity and CFD output (gas velocity) which is present in DEFIS learning (in left figure) and comparison of predicted gas velocity and CFD output (gas velocity) which is absent in DEFIS learning (in right figure) (with considering inputs 1 and 3). (**c**) Comparison of predicted gas velocity and CFD output (gas velocity) which is present in DEFIS learning (in left figure) and comparison of predicted gas velocity and CFD output (gas velocity) which is absent in DEFIS learning (in right figure) (with considering inputs 2 and 3).

**Figure 7:**
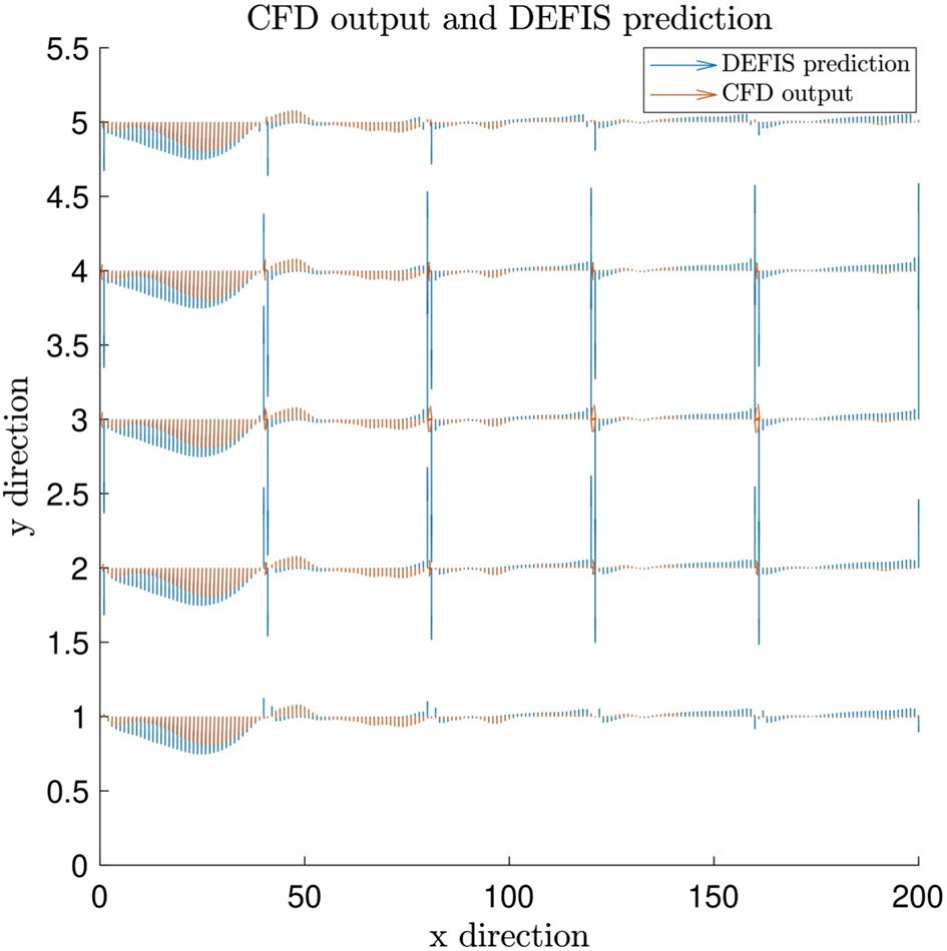
Comparison of gas velocity between predicted output via DEFIS and CFD output, x direction is number of data (node) and y direction is different heights.

The implemented data in the learning processes were extracted when the velocity of injected gas from bottom of the reactor was 0.05 and 0.11 (m/s). Furthermore, in this work by implementing FIS structure that is combined with DE algorithm in training process, extracted data from situation that the velocity of injected gas from bottom of reactor was 0.08 (m/s) was predicted. Predicted new data was compared with the previous predicted data, which is depicted in Fig. 6a–c, the left side of Fig. 6 show comparison of predicted data with the included CFD data in the learning processes while the right side of Fig. 6 show comparison of predicted new data with the CFD new data that were absent in the learning processes. As can be clearly seen from Fig. 7, there is an adaptation between vectors of predicted gas phase velocity in x direction as the output of artificial intelligence and vectors of absent CFD data in the learning processes.

**Figure 8:**
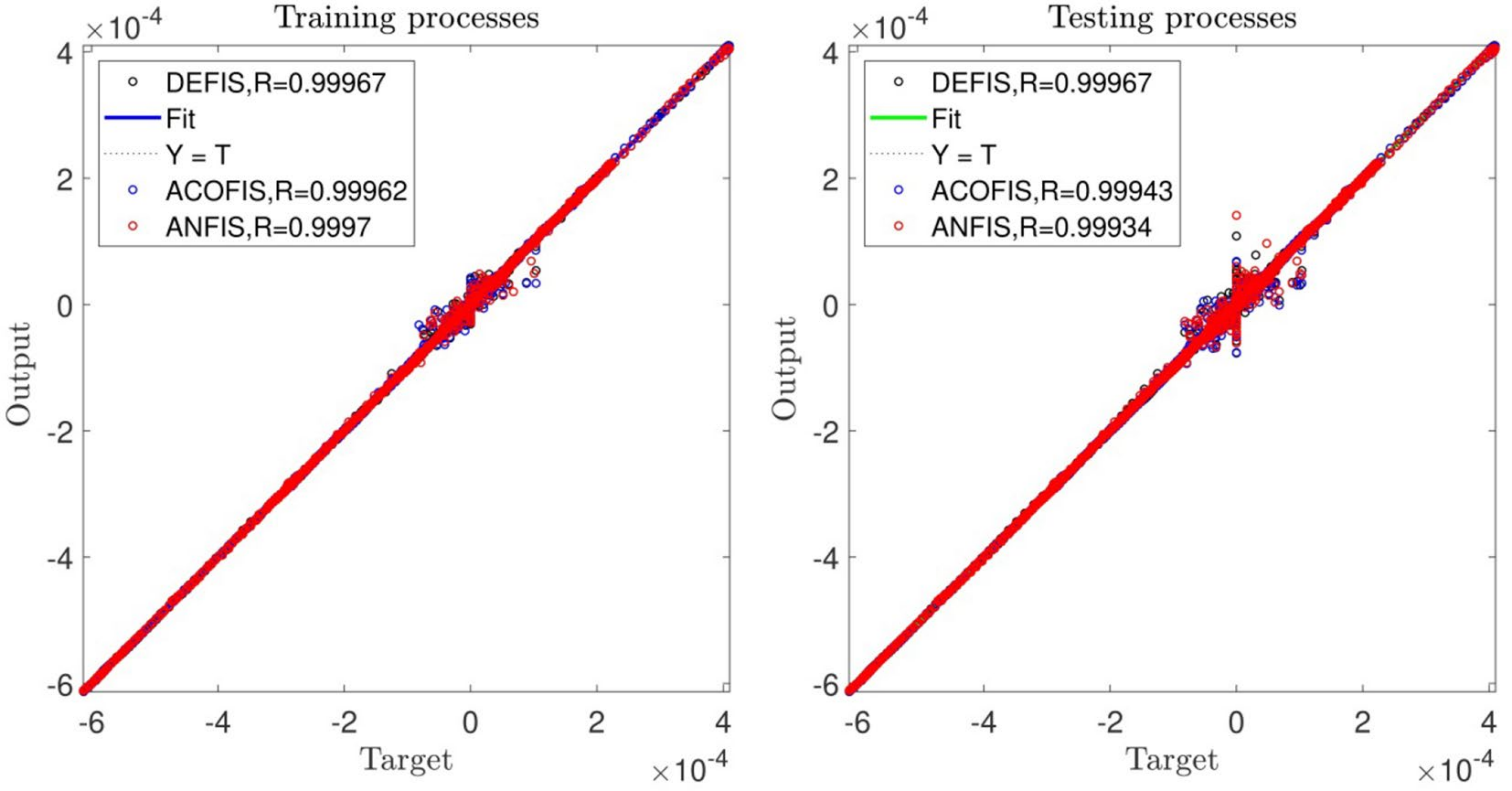
Correlation coefficient of DEFIS, ACOFIS, and ANFIS methods for training and testing processes after achieving the best intelligence.

Figure 8 shows the best system in terms of the error. We study the system with the CIR number of 0.2 and the number of population at its highest level. Also, we study the DE model with other methods, including the ANFIS and ant optimization methods, to see how the error of the system could be similar to other conventional AI models. The figure shows the regression of the system and indicates the DE model has a good prediction, which is very similar to the ACO method. The methods can predict the dataset, and the highest amount of R^2^ is achieved, which is 0.99.

**Figure 9:**
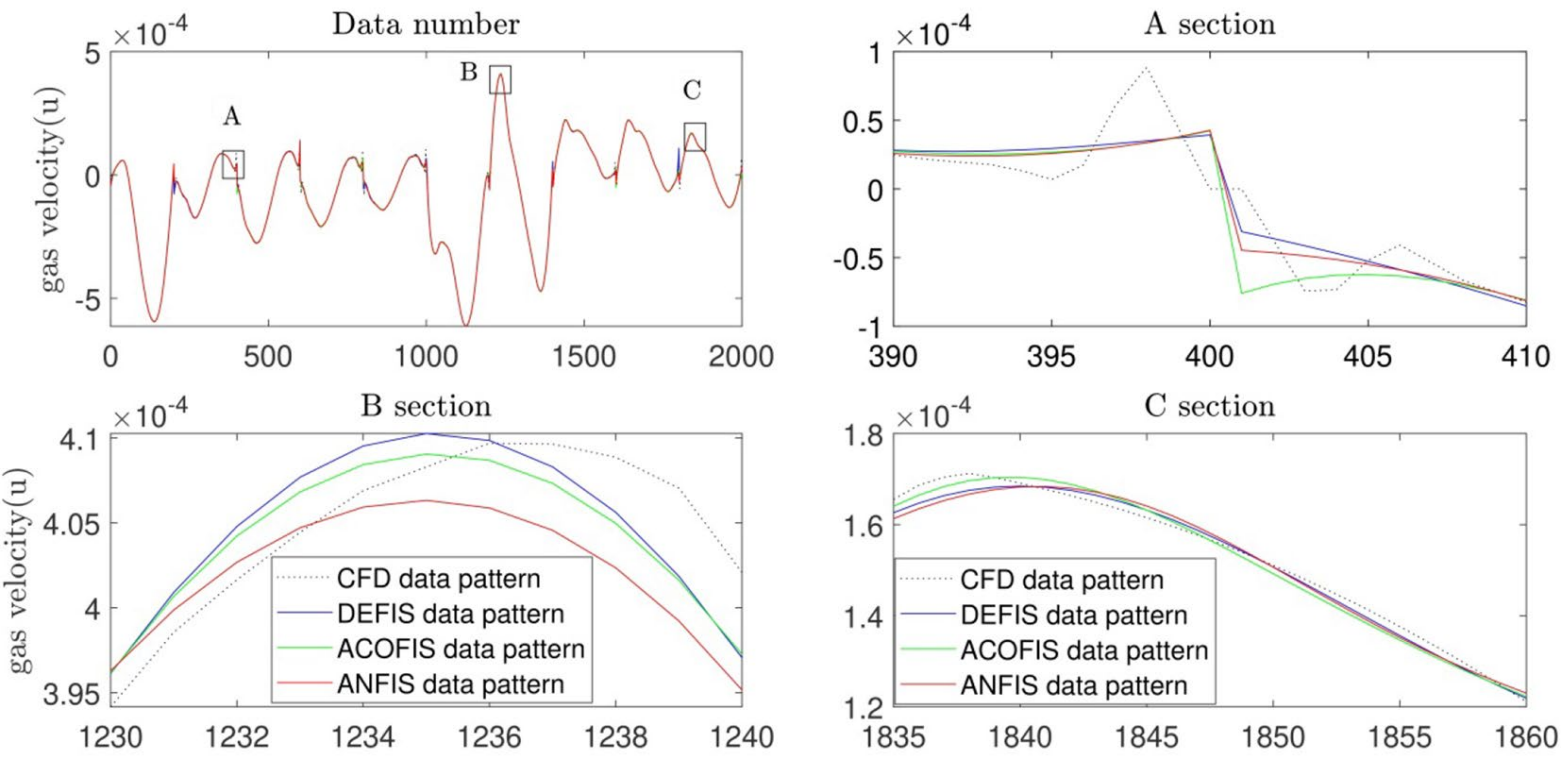
Pattern recognition for different methods such as ANFIS, DEFIS and ACOFIS.

After studying the regression of the system, we consider the pattern recognition because the accuracy and exactness of the regression of RMSE error and average error are not exact criteria for the prediction of a model. We need to study the flow and CFD patterns point by point to see how the model could be predicted. To do so, we used ANFIS and ACO methods to compare their ability with the DE model. As shown in Fig. 9, the DE model could suitably predict the gas velocity pattern for all of the numbers of data, and the flow pattern matches with CFD dataset. Moreover, the pattern is also very similar to CFD and ACO. In general, the method of DE contains a good ability of flow characteristics prediction and gas–liquid flow patterns. This method is also very similar to ANFIS and ACO with regards to pattern prediction in the domain.

**Figure 10:**
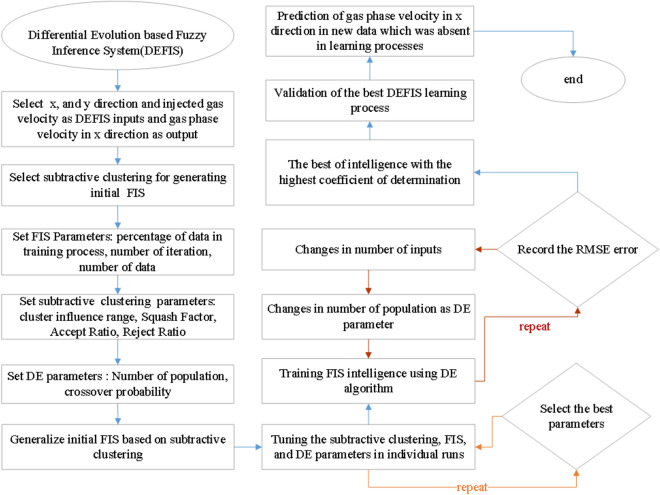
Flowchart of DEFIS method.

To fully illustrate the main flow chart of the current methodology (algorithm) and show how model parameters can impact on the final level of model accuracy and prediction capability, the main flow chart of this research is shown in Fig. 10. Figure 10 shows that x and y computing nodes and inlet gas velocity (gas sparge into the reactor) are used as input training, and gas velocity distribution as an output dataset. In the second level of the model, the FIS structure is selected based on subtractive clustering for the decision part of the model. As an initial point of running model number of iteration (epoch number), the percentage of gathering datasets and the total number of data are selected in the algorithm. Then to generate the subtractive clustering framework, several parameters are selected, such as cluster influence range, squash factor, aspect ratio, and reject ratio. After the development of subtractive clustering mode, DE parameters (number of population and crossover probability) can be defined in the model structure. We can also generalize the initial FIS structure. Then the tuning part is activated for FIS, subtractive clustering, and DE parameters. In this stage of the model, the code is evaluated for the high level of accuracy and prediction capability. Then the training campaign is started, while the RSME error is fully conducted to evaluate the high level of accuracy. In each step, if the RSME value cannot present the accurate model, the model is changed with other model parameters. In this part of the code, the number of population and number of input parameters can be changed to get the best level of accuracy and prediction capability. Then the final stage of code is activated to assess the level of accuracy, validate the model, and compare the model in predicting “*non-training*” datasets.

## Conclusions

In this study, prediction of gas phase velocity in the reactor via a combination of DE algorithm and Capacity of FIS was studied and changes in DE algorithm and FIS parameters were examined. Learning data was extracted when injected gas velocity in bottom of the reactor was 0.05 and 0.11 (m/s). By Capacity of FIS , we could completely predict gas phase velocity in x direction when injected gas velocity was between 0.05 and 0.11 (m/s) eventually 0.08 was selected and prediction process was done which shows capability of the artificial intelligence in predicting gas-phase velocity in different amount of injected gas velocity in bottom of the reactor. By comparing different types of clustering data including grid partition, subtractive and fuzzy c-mean for achieving the highest percentage of intelligence is appreciable in the future study. Comparing several algorithms (such as ANFIS Ant Colony and DE), we can conclude that the DE algorithms have high potentiality in predicting the CFD data. This algorithm can suitably complete the training process; moreover, it can complete the prediction process when the fuzzy logic system also exists in the system. Also, this algorithm can be used in pattern recognition that can match the prediction data in the target data or CFD data. We can see that CFD and DE patterns match each other*.* For future studies, using other algorithms in the training that have high potentiality seems to be advantageous. They can also have high potentiality in speeding up the learning process; therefore, we must examine them to find the best and fastest algorithm in the learning process. These algorithms can be used in different physical conditions, and their potentiality could be examined in a wide range of applications.

## Supplementary Information

Supplementary Tables.
