# Supplementary material for: Prediction of gas velocity in two-phase flow using developed fuzzy logic system with differential evolution algorithm
Source: Sci Rep. 2021 Jan 27;11:2380. doi: 10.1038/s41598-021-81957-3 (PMC7840922; doi:10.1038/s41598-021-81957-3)
Supplement: Supplementary file 1 — Supplementary Tables. [file 41598_2021_81957_MOESM1_ESM.docx]

**Prediction of gas velocity in two-phase flow using developed fuzzy logic system with differential evolution algorithm**

Meisam Babanezhad^1,2,3^, Samyar Zabihi^4^, Iman Behroyan^5,6^, Ali Taghvaie Nakhjiri^7^, Azam Marjani^8,9,*^, Saeed Shirazian^10^

^1^ Institute of Research and Development, Duy Tan University, Da Nang 550000, Vietnam

^2^ Faculty of Electrical – Electronic Engineering, Duy Tan University, Da Nang 550000, Vietnam

^3^ Department of Artificial Intelligence, Shunderman Industrial Strategy Co. Tehran, Iran

^4^ Department of Process Engineering, Research and Development Department, Shazand-Arak Oil Refinery Company, Arak, Iran

^5^ Faculty of Mechanical and Energy Engineering, Shahid Beheshti University, Tehran, Iran

^6^ Department of Computational Fluid Dynamics, Shunderman Industrial Strategy Co. Tehran, Iran

^7^ Department of Petroleum and Chemical Engineering, Science and Research Branch, Islamic Azad University, Tehran, Iran

^8^ Department for Management of Science and Technology Development, Ton Duc Thang University, Ho Chi Minh City, Viet Nam

^9^ Faculty of Applied Sciences, Ton Duc Thang University, Ho Chi Minh City, Viet Nam

^10^ Laboratory of Computational Modeling of Drugs, South Ural State University, 76 Lenin prospekt, 454080 Chelyabinsk, Russia

^*^Corresponding author; E-mail: [azam.marjani@tdtu.edu.vn](mailto:azam.marjani@tdtu.edu.vn)

**Appendix**

Table S1: DEFIS learning processes with considering diversity of cluster influence range (CIR) and number of population while number of inputs is one.

| **number of Inputs** | 1 | | | | | | | | | | | | | | | |
| --- | --- | --- | --- | --- | --- | --- | --- | --- | --- | --- | --- | --- | --- | --- | --- | --- |
| **CIR** | 0.2 | 0.2 | 0.2 | 0.2 | 0.3 | 0.3 | 0.3 | 0.3 | 0.4 | 0.4 | 0.4 | 0.4 | 0.5 | 0.5 | 0.5 | 0.5 |
| **number of population** | 4 | 8 | 12 | 16 | 4 | 8 | 12 | 16 | 4 | 8 | 12 | 16 | 4 | 8 | 12 | 16 |
| **Max Iteration** | 150 | | | | | | | | | | | | | | | |
| **P (%)** | 65 | | | | | | | | | | | | | | | |
| **clustering Type** | Subtractive clustering | | | | | | | | | | | | | | | |
| **Training Error MSE** | 3.736E-08 | 3.689E-08 | 3.772E-08 | 3.687E-08 | 3.73E-08 | 3.57E-08 | 3.74E-08 | 3.74E-08 | 3.74E-08 | 3.75E-08 | 3.57E-08 | 3.82E-08 | 3.67E-08 | 3.74E-08 | 3.7E-08 | 3.76356E-08 |
| **Training Error RMSE** | 0.0001933 | 0.0001921 | 0.0001942 | 0.000192 | 0.000193 | 0.000189 | 0.000193 | 0.000193 | 0.000193 | 0.000194 | 0.000189 | 0.000196 | 0.000191 | 0.000193 | 0.00019 | 0.000193999 |
| **Training Error Mean** | -4.899E-12 | 9.147E-14 | -2.237E-13 | -2.331E-14 | -4E-18 | 4.53E-17 | -3.4E-14 | 1.33E-14 | -6.3E-19 | -3.1E-17 | -2.3E-17 | -7.3E-18 | 1.91E-17 | -1.2E-17 | -2E-17 | 2.96005E-17 |
| **Training Error STD** | 0.0001934 | 0.0001921 | 0.0001943 | 0.0001921 | 0.000193 | 0.000189 | 0.000193 | 0.000194 | 0.000193 | 0.000194 | 0.000189 | 0.000196 | 0.000192 | 0.000193 | 0.00019 | 0.000194074 |
| **Testing Error MSE** | 3.768E-08 | 3.785E-08 | 3.784E-08 | 3.781E-08 | 3.76E-08 | 3.77E-08 | 3.77E-08 | 3.76E-08 | 3.76E-08 | 3.76E-08 | 3.76E-08 | 3.77E-08 | 3.76E-08 | 3.76E-08 | 3.8E-08 | 3.756E-08 |
| **Testing Error RMSE** | 0.0001941 | 0.0001946 | 0.0001945 | 0.0001944 | 0.000194 | 0.000194 | 0.000194 | 0.000194 | 0.000194 | 0.000194 | 0.000194 | 0.000194 | 0.000194 | 0.000194 | 0.00019 | 0.000193804 |
| **Testing Error Mean** | -8.659E-07 | 5.687E-06 | -1.943E-07 | -3.108E-06 | 2.9E-06 | -4.3E-06 | 2.95E-07 | -2.1E-07 | -1.6E-06 | 1.65E-06 | -2.3E-06 | -2.2E-06 | -4.2E-06 | -2.7E-06 | -9E-07 | 3.29741E-06 |
| **Test Error STD** | 0.0001942 | 0.0001945 | 0.0001946 | 0.0001945 | 0.000194 | 0.000194 | 0.000194 | 0.000194 | 0.000194 | 0.000194 | 0.000194 | 0.000194 | 0.000194 | 0.000194 | 0.00019 | 0.000193825 |

Table S2: DEFIS learning processes with considering diversity of cluster influence range (CIR) and number of population while number of inputs is two.

| **number of inputs** | 2 | | | | | | | | | | | | | | | |
| --- | --- | --- | --- | --- | --- | --- | --- | --- | --- | --- | --- | --- | --- | --- | --- | --- |
| **CIR** | 0.2 | 0.2 | 0.2 | 0.2 | 0.3 | 0.3 | 0.3 | 0.3 | 0.4 | 0.4 | 0.4 | 0.4 | 0.5 | 0.5 | 0.5 | 0.5 |
| **number of population** | 4 | 8 | 12 | 16 | 4 | 8 | 12 | 16 | 4 | 8 | 12 | 16 | 4 | 8 | 12 | 16 |
| **Max Iteration** | 150 | | | | | | | | | | | | | | | |
| **number of P** | 65 | | | | | | | | | | | | | | | |
| **clustering Type** | Subtractive clustering | | | | | | | | | | | | | | | |
| **Train Error MSE** | 1.61E-08 | 1.63E-08 | 1.54E-08 | 1.62E-08 | 1.71E-08 | 1.68E-08 | 1.69E-08 | 1.64E-08 | 1.64E-08 | 1.66E-08 | 1.72E-08 | 1.71E-08 | 1.71E-08 | 1.75E-08 | 1.76E-08 | 1.84E-08 |
| **Train Error RMSE** | 0.000127 | 0.000128 | 0.000124 | 0.000127 | 0.000131 | 0.000129 | 0.00013 | 0.000128 | 0.000128 | 0.000129 | 0.000131 | 0.000131 | 0.000131 | 0.000132 | 0.000133 | 0.000136 |
| **Train Error Mean** | 2.32E-11 | 4.45E-12 | 5.8E-11 | -1.7E-10 | 7.73E-12 | 7.39E-15 | -8.5E-15 | -9.2E-15 | -6.6E-14 | 8.26E-17 | 3.96E-18 | 1.33E-17 | -8.8E-20 | 1.86E-20 | 2.56E-19 | -9.1E-21 |
| **Train Error STD** | 0.000127 | 0.000128 | 0.000124 | 0.000127 | 0.000131 | 0.00013 | 0.00013 | 0.000128 | 0.000128 | 0.000129 | 0.000131 | 0.000131 | 0.000131 | 0.000132 | 0.000133 | 0.000136 |
| **Test Error MSE** | 1.78E-08 | 1.76E-08 | 1.79E-08 | 1.77E-08 | 1.73E-08 | 1.72E-08 | 1.73E-08 | 1.74E-08 | 1.73E-08 | 1.71E-08 | 1.71E-08 | 1.71E-08 | 1.75E-08 | 1.77E-08 | 1.73E-08 | 1.79E-08 |
| **Test Error RMSE** | 0.000133 | 0.000133 | 0.000134 | 0.000133 | 0.000132 | 0.000131 | 0.000131 | 0.000132 | 0.000131 | 0.000131 | 0.000131 | 0.000131 | 0.000132 | 0.000133 | 0.000131 | 0.000134 |
| **Test Error Mean** | 1.26E-06 | -4.8E-06 | -2.4E-06 | 1.7E-06 | -2E-06 | 4.35E-07 | 1.15E-06 | 2.11E-06 | 3.48E-06 | -3.3E-07 | -3.9E-06 | -2.3E-06 | 1.95E-06 | 7.53E-07 | 9.56E-08 | 1.27E-06 |
| **Test Error STD** | 0.000133 | 0.000133 | 0.000134 | 0.000133 | 0.000132 | 0.000131 | 0.000131 | 0.000132 | 0.000131 | 0.000131 | 0.000131 | 0.000131 | 0.000132 | 0.000133 | 0.000131 | 0.000134 |

Table S3: DEFIS learning processes with considering diversity of cluster influence range(CIR) and number of population while number of inputs is three.

| **number of inputs** | 3 | | | | | | | | | | | | | | | |
| --- | --- | --- | --- | --- | --- | --- | --- | --- | --- | --- | --- | --- | --- | --- | --- | --- |
| **CIR** | 0.2 | 0.2 | 0.2 | 0.2 | 0.3 | 0.3 | 0.3 | 0.3 | 0.4 | 0.4 | 0.4 | 0.4 | 0.5 | 0.5 | 0.5 | 0.5 |
| **number of population** | 4 | 8 | 12 | 16 | 4 | 8 | 12 | 16 | 4 | 8 | 12 | 16 | 4 | 8 | 12 | 16 |
| **Max Iteration** | 150 | | | | | | | | | | | | | | | |
| **number of P** | 65 | | | | | | | | | | | | | | | |
| **clustering Type** | Subtractive clustering | | | | | | | | | | | | | | | |
| **Train Error MSE** | 3.66E-11 | 3.36E-11 | 2.9E-11 | 2.71E-11 | 4.08E-11 | 3.84E-11 | 4.09E-11 | 5.52E-11 | 1.29E-10 | 1.79E-10 | 1.51E-10 | 1.62E-10 | 7.05E-10 | 4.38E-10 | 6.73E-10 | 2.22E-10 |
| **Train Error RMSE** | 6.05E-06 | 5.8E-06 | 5.39E-06 | 5.2E-06 | 6.39E-06 | 6.2E-06 | 6.39E-06 | 7.43E-06 | 1.14E-05 | 1.34E-05 | 1.23E-05 | 1.27E-05 | 2.66E-05 | 2.09E-05 | 2.59E-05 | 1.49E-05 |
| **Train Error Mean** | -9.5E-15 | -1.1E-12 | -2.3E-15 | -7.7E-16 | -2.4E-13 | -1.2E-11 | -8.5E-16 | -3.2E-17 | -5.7E-18 | -1.1E-18 | 1.32E-11 | 7.84E-19 | -3.5E-12 | -5.2E-12 | 3.26E-12 | 5.64E-12 |
| **Train Error STD** | 6.05E-06 | 5.8E-06 | 5.39E-06 | 5.2E-06 | 6.39E-06 | 6.2E-06 | 6.4E-06 | 7.44E-06 | 1.14E-05 | 1.34E-05 | 1.23E-05 | 1.27E-05 | 2.66E-05 | 2.09E-05 | 2.59E-05 | 1.49E-05 |
| **Test Error MSE** | 4.95E-11 | 5.15E-11 | 6.21E-11 | 5.26E-11 | 5.22E-11 | 4.99E-11 | 5.12E-11 | 5.3E-11 | 1.88E-10 | 1.74E-10 | 1.59E-10 | 1.7E-10 | 6.9E-10 | 4.22E-10 | 6.96E-10 | 2.46E-10 |
| **Test Error RMSE** | 7.04E-06 | 7.17E-06 | 7.88E-06 | 7.25E-06 | 7.23E-06 | 7.07E-06 | 7.16E-06 | 7.28E-06 | 1.37E-05 | 1.32E-05 | 1.26E-05 | 1.3E-05 | 2.63E-05 | 2.05E-05 | 2.64E-05 | 1.57E-05 |
| **Test Error Mean** | 5.48E-08 | 2.14E-07 | -2.7E-07 | -1.8E-07 | -2.8E-08 | 7.7E-08 | -2.7E-07 | -4.8E-08 | 5.48E-07 | -4.8E-07 | 3.48E-07 | -3.5E-07 | 5.39E-08 | 6.71E-08 | 5E-08 | 2.91E-08 |
| **Test Error STD** | 7.04E-06 | 7.17E-06 | 7.87E-06 | 7.25E-06 | 7.23E-06 | 7.07E-06 | 7.15E-06 | 7.28E-06 | 1.37E-05 | 1.32E-05 | 1.26E-05 | 1.3E-05 | 2.63E-05 | 2.06E-05 | 2.64E-05 | 1.57E-05 |
